# Supplementary material for: Dipolar-stabilized first and second-order antiskyrmions in ferrimagnetic multilayers
Source: Nat Commun. 2021 May 10;12:2611. doi: 10.1038/s41467-021-22600-7 (PMC8110839; doi:10.1038/s41467-021-22600-7)
Supplement: Supplementary file 1 — Supplementary Information [file 41467_2021_22600_MOESM1_ESM.pdf]

Supplementary information - Dipolar-stabilized first and second-order antiskyrmions in ferrimagnetic multilayers

Michael Heigl[1*], Sabri Koraltan[2], Marek Vaňatka[3], Robert Kraft[2], Claas Abert[2,4], Christoph Vogler[2], Anna Semisalova[5], Ping Che[6], Aladin Ullrich[1], Timo Schmidt[1], Julian Hintermayr[1], Dirk Grundler[6], Michael Farle[5], Michal Urbánek[3], Dieter Suess[2,4], and Manfred Albrecht[1]
[1] Institute of Physics, University of Augsburg, Augsburg 86159, Germany
[2] Faculty of Physics, University of Vienna, Vienna 1090, Austria
[3] CEITEC BUT, Brno University of Technology, Brno 61200, Czech Republic
[4] Research Platform MMM Mathematics - Magnetism - Materials, University of Vienna, Vienna 1090, Austria
[5] Center for Nanointegration and Faculty of Physics, University of Duisburg-Essen, Duisburg 47057, Germany
[6] École Polytechnique Fédérale de Lausanne, School of Engineering, Institute of Materials, Lausanne 1015, Switzerland
Email: * michael.heigl@uni-a.de

SQUID-VSM measurements. All measured $M-H$ hysteresis loops of the [Fe(0.35)/Ir(0.20)/Gd(0.40)]$_{N_{\mathrm{Ir}}}$ / [Fe(0.35)/Gd(0.40)]$_{80-2N_{\mathrm{Ir}}}$ / [Fe(0.35)/Ir(0.20)/Gd(0.40)]$_{N_{\mathrm{Ir}}}$ MLs in oop and ip geometry for temperatures between 50 and 350 K are displayed in Fig. 9. Due to the decrease in magnetization with temperature, we conclude that all Fe/Gd-based MLs are Gd dominant over all measured temperatures. The formation of spin textures is sometimes revealed by the presence of an opening in the oop loop close to saturation.

Figure 10 shows the dependence of the saturation magnetization M_{s} (a) and the uniaxial magnetic anisotropy K_{u} (b) on N_{Ir}. The error bars account for uncorrected geometry effects of the samples during SQUID measurements (around ±10%). The insertion of Ir layers reduces linearly the saturation magnetization (Fig. 10 a). If all 80 layers have additional Ir insertion layers, M_{s} almost vanishes completely (N_{Ir}=40). Thus, we conclude that Ir insertion layers reduce the total moment of the adjacent Fe and Gd layers to nearly zero. It is also important to note that while the moment decreases with the increasing number of Ir insertion layers, the shape of the loops stays relatively similar due to the approximately constant ratio of magnetic shape and magnetic uniaxial anisotropy. Furthermore, all samples reveal rather small K_{u} values, which decrease with increasing N_{Ir}, as summarized in Fig. 10 b for different temperatures.

Further phase diagrams. Phase diagrams of the discussed sample series dependent on the oop magnetic field and temperature are displayed in Fig. 11. No pure antiskyrmion phase is observable. Antiskyrmions always coexist with Bloch skyrmions and sometimes with type-2 bubbles. It is also evident that the stability range of antiskyrmions is smaller than the one of skyrmions for both temperature and field for all samples. For five of the six samples, it was possible to stabilize antiskyrmions even at room temperature. While we observed first-order antiskyrmions in every sample of our series besides $N_{\mathrm{Ir}}=40$ at a wide range of temperatures, second-order antiskyrmions were only observed at 260 K in $N_{\mathrm{Ir}}=2$ (Fig. 11 b) and at 300 K in $N_{\mathrm{Ir}}=20$ (Fig. 11 e). In all cases, they were quite rare in comparison to the other spin objects. Because of that, we can not rule out their existence at other temperatures and fields. For $N_{\mathrm{Ir}}=40$ no magnetic LTEM contrast was observable due to the low magnetization of the sample (see Fig. 11 f).

Magnetic force microscopy image. To confirm the underlying magnetic periodicity of our samples, magnetic force microscopy images were captured. Figure 12 shows the LTEM image (Fig. 12 a) of [Fe/Gd]$_{80}$ in comparison to an exemplary MFM image (Fig. 12 b). Both images were captured in zero field at room temperature. Note that the images do not show the same region of the sample but share the same scale. The size of the magnetic domains captured by MFM match roughly the size of the larger high-contrast black and white stripes in the defocused LTEM image.

Additional LTEM images. Some additional LTEM images of selected samples at various temperatures and oop magnetic fields are shown in Fig. 13. Figure 13 a-d displays LTEM images of the [Fe/Ir/Gd]$_2$ / [Fe/Gd]$_{76}$ / [Fe/Ir/Gd]$_2$ ML at 260 K. Image a) is captured in a magnetic field of 92 mT and shows first and second-order antiskyrmions, counterclockwise and clockwise Bloch skyrmions, type-2 bubbles, and stripes with and without chirality. In image b) all stripes shrunk down to circular spin textures. An image of this field sweep is displayed in Fig. 1 a of the main text. At 115 mT type-2 bubbles and first-order antiskyrmions have vanished. Only

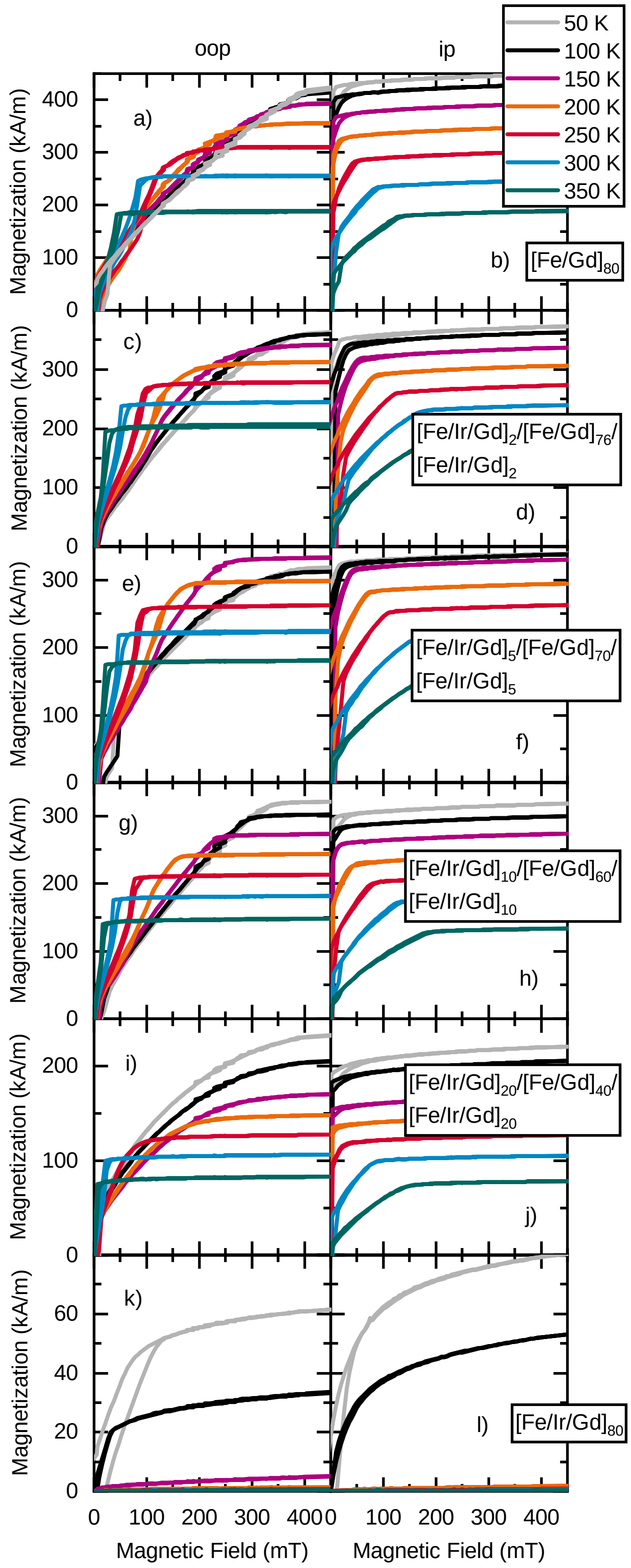

Fig. 9. **$M - H$ hysteresis loops.** $M - H$ hysteresis loops of [Fe(0.35)/Ir(0.20)/Gd(0.40)]$_{N_{\mathrm{Ir}}}$ / [Fe(0.35)/Gd(0.40]$_{80-2N_{\mathrm{Ir}}}$ / [Fe(0.35)/Ir(0.20)/Gd(0.40)]$_{N_{\mathrm{Ir}}}$ MLs in oop and ip geometry for temperatures at 50, 100, 150, 200, 250, 300, and 350 K. N_{Ir} = 0 (a, b), 2 (c, d), 5 (e, f), 10 (g, h), 20 (i, J), 40 (k, l) are displayed.

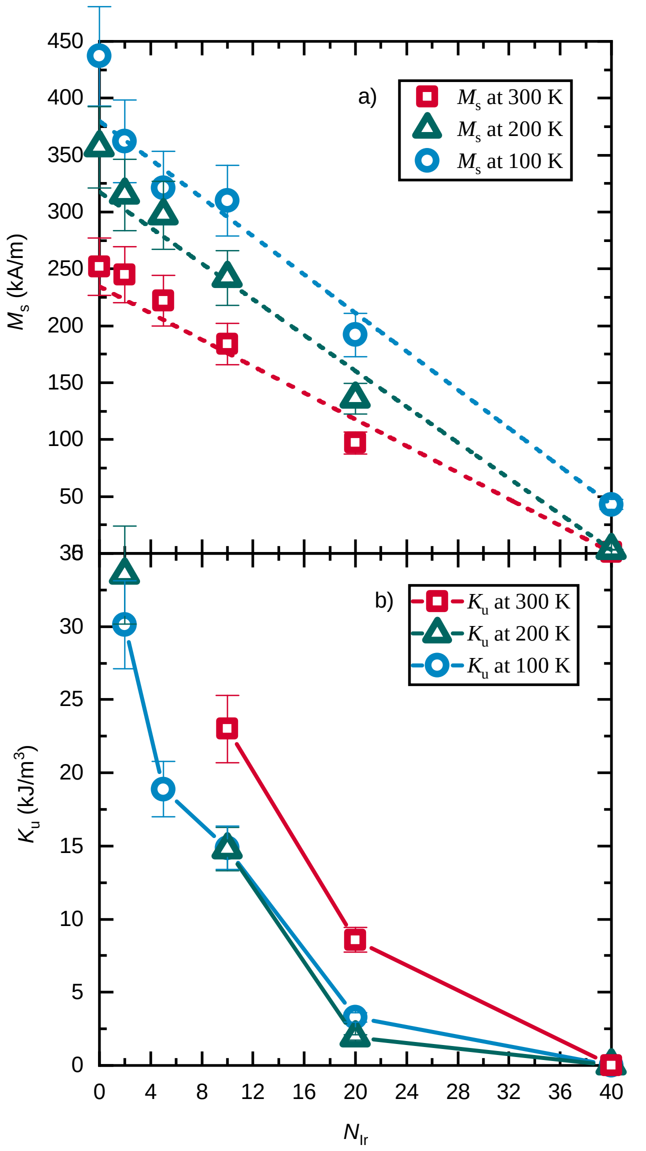

Fig. 10. **Magnetic values.** a) Saturation magnetization M_s and b) uniaxial anisotropy K_u as a function of repetition number N_{Ir} of Ir insertion layers.

the second-order antiskyrmion in the center of the image and Bloch skyrmions are left (c). Image d) shows only Bloch skyrmions at 127 mT. Figure 13 e-h shows LTEM images of the [Fe/Ir/Gd]$_5$ / [Fe/Gd]$_{70}$ / [Fe/Ir/Gd]$_5$ ML at 300 K. Image e) displays the ground state at zero field. This sample exhibits only stripes with chirality at 300 K. In image f) Bloch skyrmions start to nucleate at 57 mT. At 70 mT also first-order antiskyrmions appear (g). In image h) exclusively skyrmions are stable at 83 mT. Figure 13 i-l displays LTEM images of the [Fe/Ir/Gd]$_5$ / [Fe/Gd]$_{70}$ / [Fe/Ir/Gd]$_5$ ML at 100 K. Skyrmions nucleate in this sample and at this temperature at 67 mT (j), while type-2 bubbles and first-order antiskyrmions appear at 115 mT (k). At 134 mT only the topologically protected (anti-)skyrmions are visible (l). Figure 13 m-p shows LTEM images of the [Fe/Ir/Gd]$_{20}$ / [Fe/Gd]$_{40}$ / [Fe/Ir/Gd]$_{20}$ ML at 300 K. The contrast is weaker for this sample because of the reduced

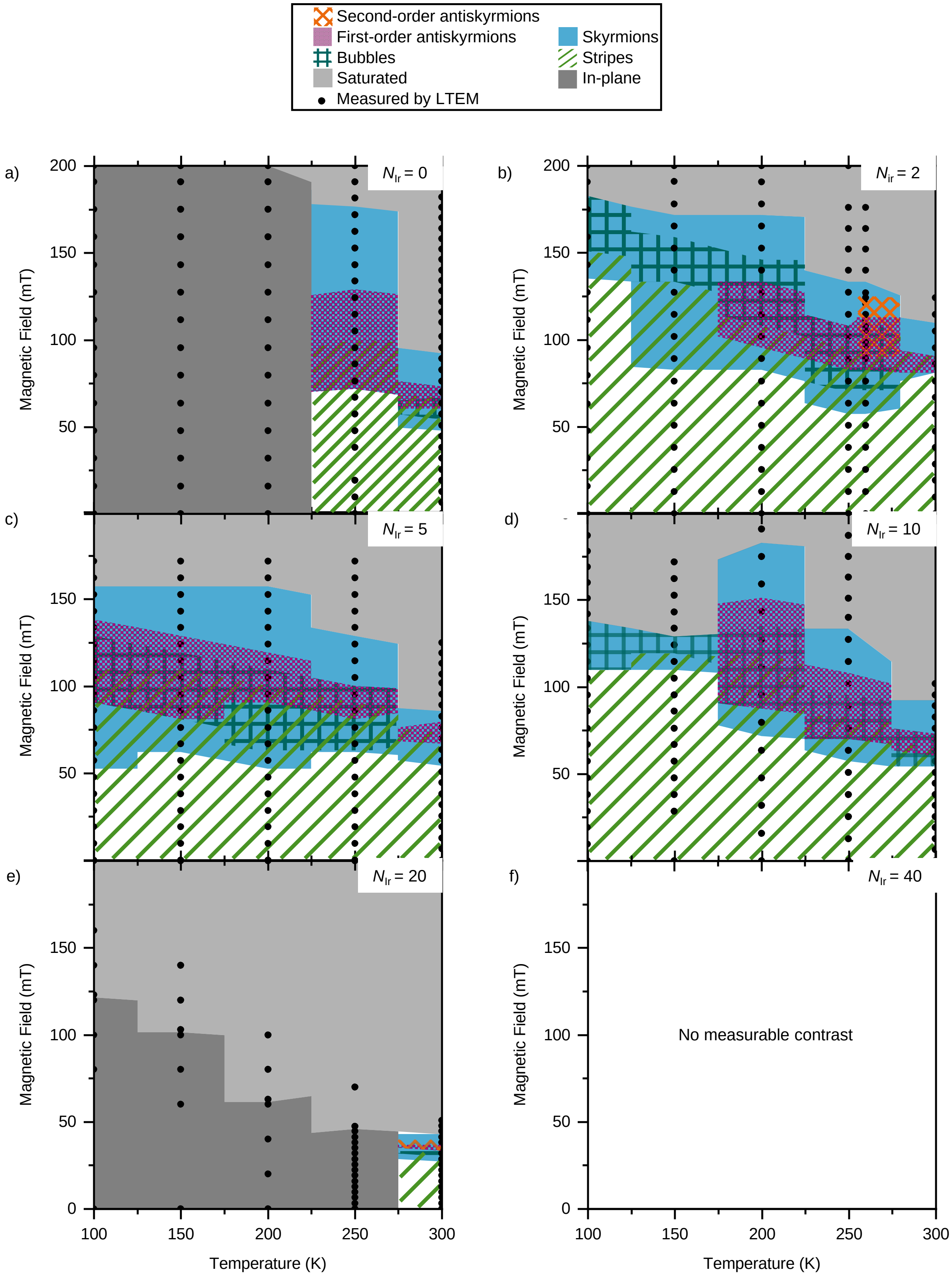

Fig. 11. **Additional phase maps.** Magnetic field and temperature dependence of the phases of the different spin structures. The magnetic phase diagrams of a) $[Fe/Gd]_{80}$, b) $[Fe/Ir/Gd]_2$ / $[Fe/Gd]_{76}$ / $[Fe/Ir/Gd]_2$, c) $[Fe/Ir/Gd]_5$ / $[Fe/Gd]_{70}$ / $[Fe/Ir/Gd]_5$, d) $[Fe/Ir/Gd]_{10}$ / $[Fe/Gd]_{60}$ / $[Fe/Ir/Gd]_{10}$, e) $[Fe/Ir/Gd]_{20}$ / $[Fe/Gd]_{40}$ / $[Fe/Ir/Gd]_{20}$, and f) $[Fe/Ir/Gd]_{80}$ are displayed.

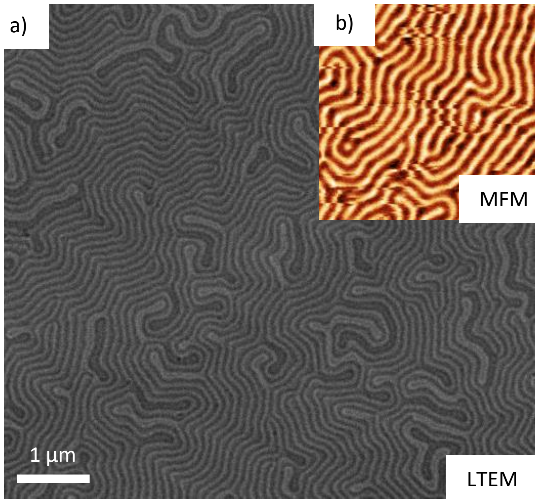

Fig. 12. **Comparison between MFM and LTEM images.** Exemplary LTEM image (a) of $[Fe/Gd]_{80}$ in comparison to the MFM image (b). Both images were captured in zero field at room temperature. Note that the images do not show the same region of the sample but share the same scale.

magnetic moment (Fig. 10 a). Although image m) exhibits only magnetic stripes with chirality at zero field, image n) shows type-2 bubbles at 32 mT. At 35 mT skyrmions and antiskyrmions start to nucleate while the bubbles vanish (o). The sample is saturated at 45 mT (p).

Micromagnetic simulations. In addition to the simulations presented in the paper, further calculations were performed.

In addition to Fig. 5, we provide in Fig. 14 the formation process of a clockwise (CW) and counterclockwise (CCW) Bloch skyrmions. Figure 15 illustrates the magnetization states at chosen magnitudes of the applied Zeeman field during the formation of a second-order antiskyrmion in a system with weak DMI using $D = 0.1\,\mathrm{mJ/m^2}$. The DMI energy is modelled in our micromagnetic simulations as

$$E_{\mathrm{DMI}} = \int_{\Omega_m} D\left[m\cdot\nabla(e_d\cdot m) - (\nabla\cdot m)(e_d\cdot m)\mathrm{d}x\right], \qquad (1)$$

where Ω_m is the magnetic region, $e_d = (0,\ 0,\ 1)$, D is the DMI constant, and m is the normalized magnetization vector.

It is noteworthy that in our micromagnetic investigations the second-order antiskyrmions only appear in the presence of weak DMI, where $D = 0.1\,\mathrm{mJ/m^2}$. However, they are really rare and we can not rule out their presence in systems without DMI.

In order to investigate the stability of skyrmions and antiskyrmions in infinite films, we simulate their magnetization dynamics in a confined system where we chose a rough parametrization as start configuration. Furthermore, to avoid the decay of the topological structure by moving out of the confined system, we set a high anisotropy of $K_u = 1\,\mathrm{MJ/m^3}$ in the outer ring of the simulated structure. If this anisotropy barrier is placed at a sufficient distance from the topological structure, this procedure is assumed to accurately account for possible annihilation processes in infinite films, while keeping the computational cost at a feasible level.

Figure 16 illustrates the obtained stability phase maps of an antiskyrmion for a system free of DMI at different Zeeman fields. To obtain these results, we apply the Zeeman fields constantly for 10 ns, while solving the LLG, Eq. 1, in a system where a parametrization of an antiskyrmion is the initial magnetic configuration. The exchange stiffness constant is chosen as constant at $A_{\mathrm{ex}} = 6\,\mathrm{pJ/m}$. The final magnetization state $m(10\,\mathrm{ns})$ is then evaluated by means of the finite element method and the integer topological charge N_{sk} is calculated integrating Eq. 2 over the entire volume, and dividing the result by the thickness of the sample, which is 62 nm.

In order to show the coexistence of the skyrmions in the regimes where an antiskyrmion is dipolar-stabilized, we repeated the micromagnetic simulations shown in Fig. 16 by using a Bloch skyrmion as the initial magnetization state. The phase maps obtained from these simulations are illustrated in Figs. 17.

To show, that the spin objects with $N_{\mathrm{sk}} = -1$ can be found only in a very restricted regime, we chose to investigate the role of the DMI in their stability. Figure 18 a shows that our experiments are exactly in the regime, where these structures can be nucleated and stabilized. By making use of a different composition of Fe/Gd-layers and the introduction Ir insertion layers, our experiments show particularly low values for M_s and K_u, an aspect of crucial importance. We see in Fig. 18 a that a DMI value higher than $D_{max} = 0.2\,\mathrm{mJ/m^2}$ leads to a system where the antiskyrmion is no longer a stable configuration, and skyrmions dominate.

Additionally, the dipolar-stabilization dominates the systems in such manner that the exchange interactions do not have any crucial influence on the stability of the antiskyrmions, as it is shown in Fig. 18 b. Nevertheless, for increasing M_s and A we notice that the integer topological charge starts to deviate from $N_{\mathrm{sk}} = -1$, as the antiskyrmions start to deform at the edges. These simulations have been performed without DMI and an uniaxial magnetic anisotropy constant $K_u = 22.35\,\mathrm{kJ/m^3}$ was used.

To show that the total thickness of the investigated samples does not influence the stability of the antiskyrmions, we vary the thickness of our magnetic material together with M_s. Further material parameters are chosen as $A = 6\,\mathrm{pJ/m}$ and $K_u = 22.35\,\mathrm{kJ/m^3}$. Figure 18 c shows the phase diagram using a system free of DMI. It is very clear that the thickness does not lead to any deformations or instabilities in the investigated thickness range.

In order to exclude the possibility that the spin textures are stable in the micromagnetic simulations because of numerical errors and simulation artifacts, we perform energy barrier simulations applying a full micromagnetic model [1–3]. Namely, we calculate the minimum energy paths to annihilate an antiskyrmion to obtain a saturated magnetic configuration along the e_z- direction and to transform an antiskyrmion in a skyrmion. The former is shown in Fig. 19 a and the latter in Fig. 19 b. Both simulations show a clear energy barrier, reflecting the metastable nature of the antiskyrmion state. It is also observed that other energetically more favorable states need to be overcome in order to transition to skyrmions or to the saturated state, making these transformations very costly

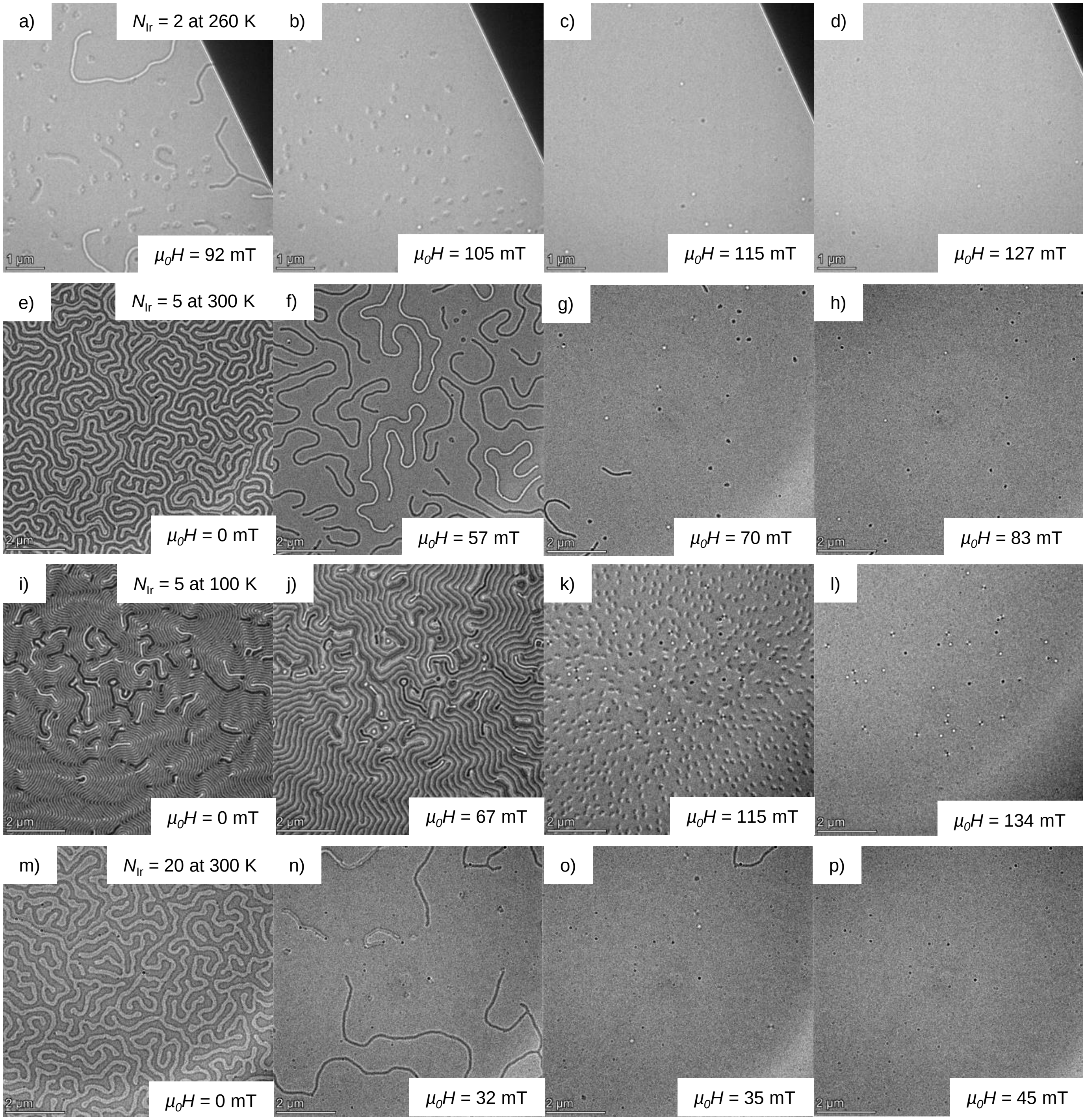

Fig. 13. **Additional LTEM images.** Exemplary LTEM images of selected samples at various temperatures and oop magnetic fields H. a-d) displays $[Fe/Ir/Gd]_2$ / $[Fe/Gd]_{76}$ / $[Fe/Ir/Gd]_2$ at 260 K. e-h) shows $[Fe/Ir/Gd]_5$ / $[Fe/Gd]_{70}$ / $[Fe/Ir/Gd]_5$ at 300 K and i-l) at 100 K. m-p) shows $[Fe/Ir/Gd]_{20}$ / $[Fe/Gd]_{40}$ / $[Fe/Ir/Gd]_{20}$ at 300 K.

from an energetic point-of-view. It has to be mentioned that, due to the change of the topological number N_{sk} from -1 to 1, Bloch like states are formed and a micromagnetic treatment with a mesh size larger than the lattice constant underestimates the atomistic barrier [4].

References

1. E, W., Ren, W. & Vanden-Eijnden, E. Simplified and improved string method for computing the minimum energy paths in barrier-crossing events. *Int. J. Chem. Phys.* **126,** 164103 (2007).
2. Abert, C. Micromagnetics and spintronics: models and numerical methods. *Eur. Phys. J. B* **92,** 120 (2019).
3. Koraltan, S. *et al.* Dependence of energy barrier reduction on collective excitations in square artificial spin ice: A comprehensive comparison of simulation techniques. *Phys. Rev. B* **102,** 064410 (2020).
4. Suess, D., Vogler, C., Bruckner, F., Heistracher, P. & Abert, C. A repulsive skyrmion chain as a guiding track for a racetrack memory. *AIP Adv.* **8,** 115301 (2018).
5. Farle, M. Ferromagnetic resonance of ultrathin metallic layers. *Rep. Prog. Phys.* **61,** 755 (1998).

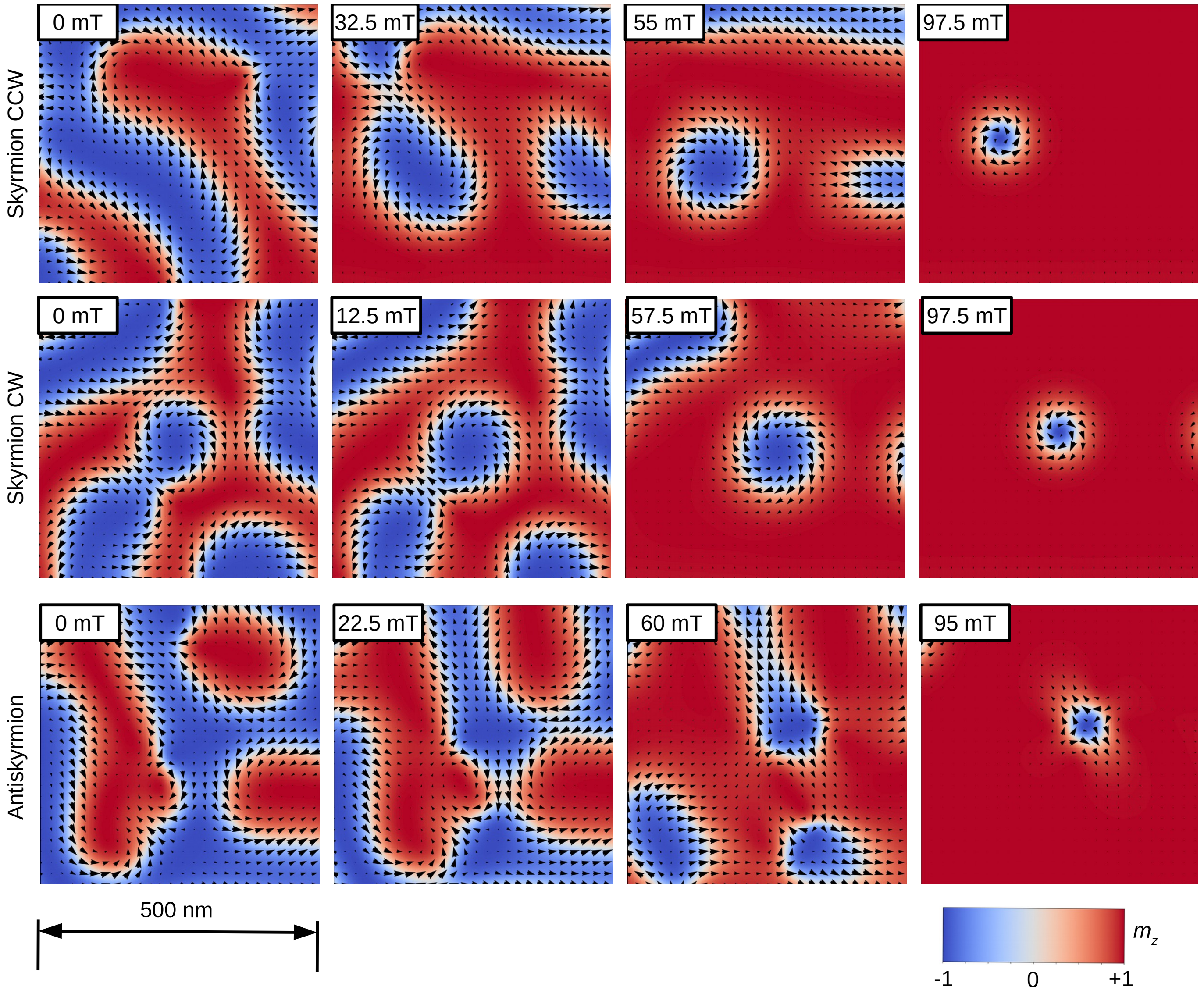

Fig. 14. **Simulated formation process of a (counter-)clockwise skyrmion and antiskyrmion without DMI.**

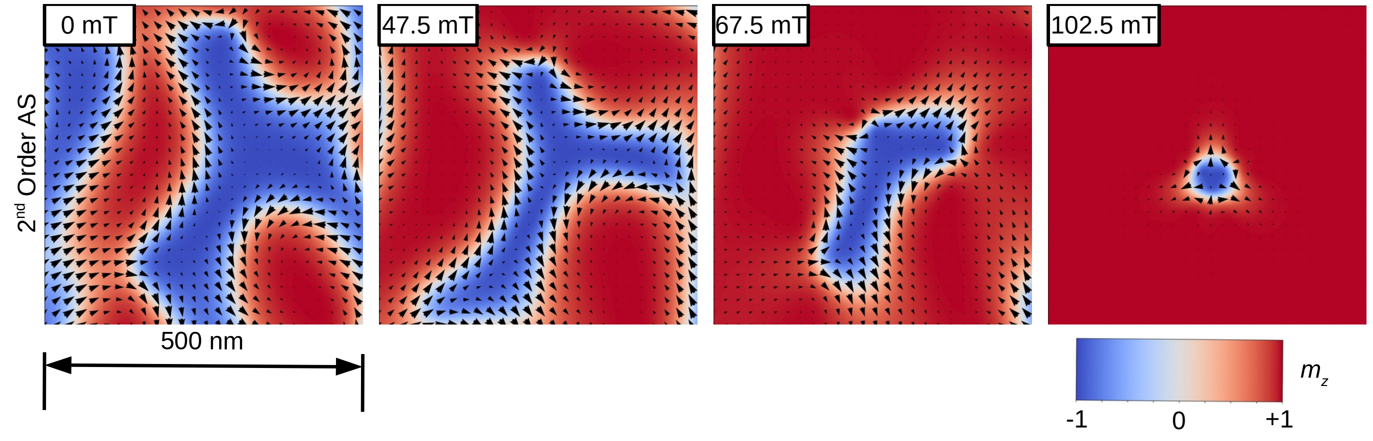

Fig. 15. **Simulated formation process of a second-order antiskyrmion in a system with weak DMI ($D = 0.1\,\mathrm{mJ/m^2}$).**

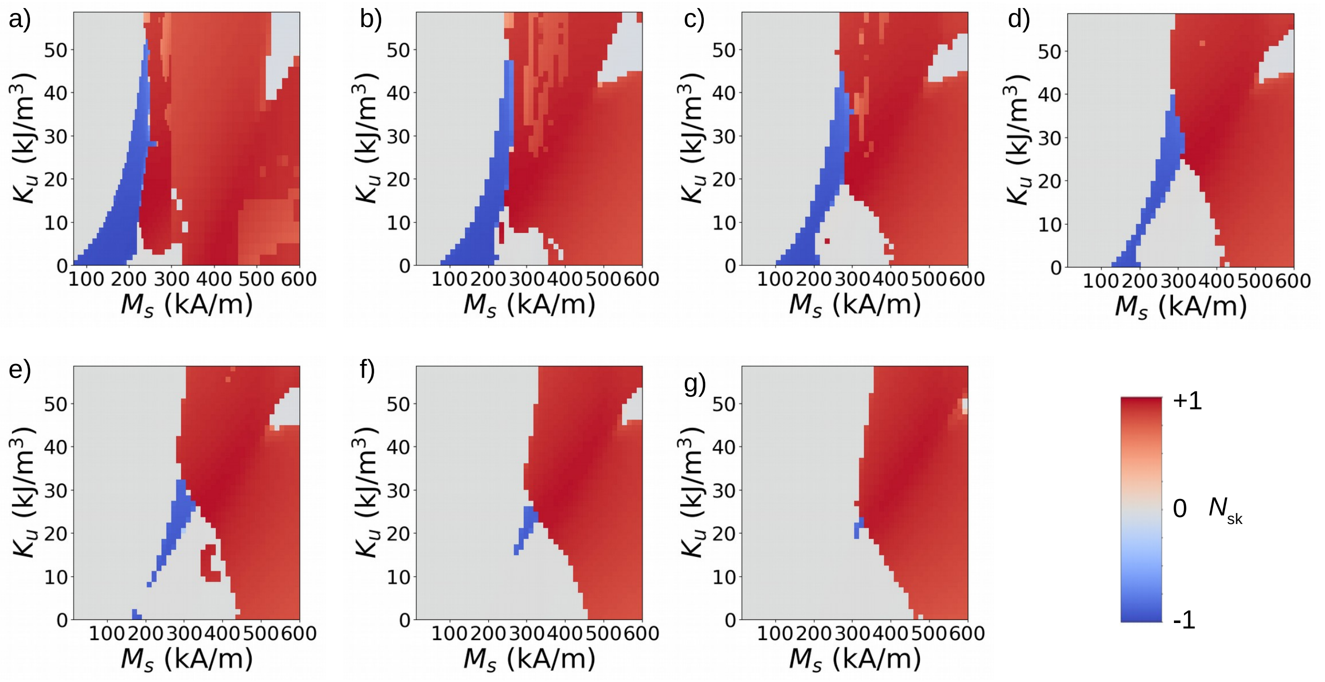

Fig. 16. **Spin object phase diagrams with an initial antiskyrmion.** Diagrams of M_s and K_u ($10\,\mathrm{kJ/m^3} \sim 0.7\,\mu\mathrm{eV/atom}$ [5]) dependent stability of an isolated antiskyrmion obtained from micromagnetic simulations without DMI and with constantly applied Zeeman fields of strength a) $\mu_0 H = 0\,\mathrm{mT}$, b) $\mu_0 H = 10\,\mathrm{mT}$, c) $\mu_0 H = 30\,\mathrm{mT}$, d) $\mu_0 H = 50\,\mathrm{mT}$, e) $\mu_0 H = 70\,\mathrm{mT}$, f) $\mu_0 H = 100\,\mathrm{mT}$, and g) $\mu_0 H = 125\,\mathrm{mT}$. The integer topological charge N_{sk} is -1 for an antiskyrmion, 1 for a skyrmion, and 0 for a magnetization state with no topologically protected spin object.

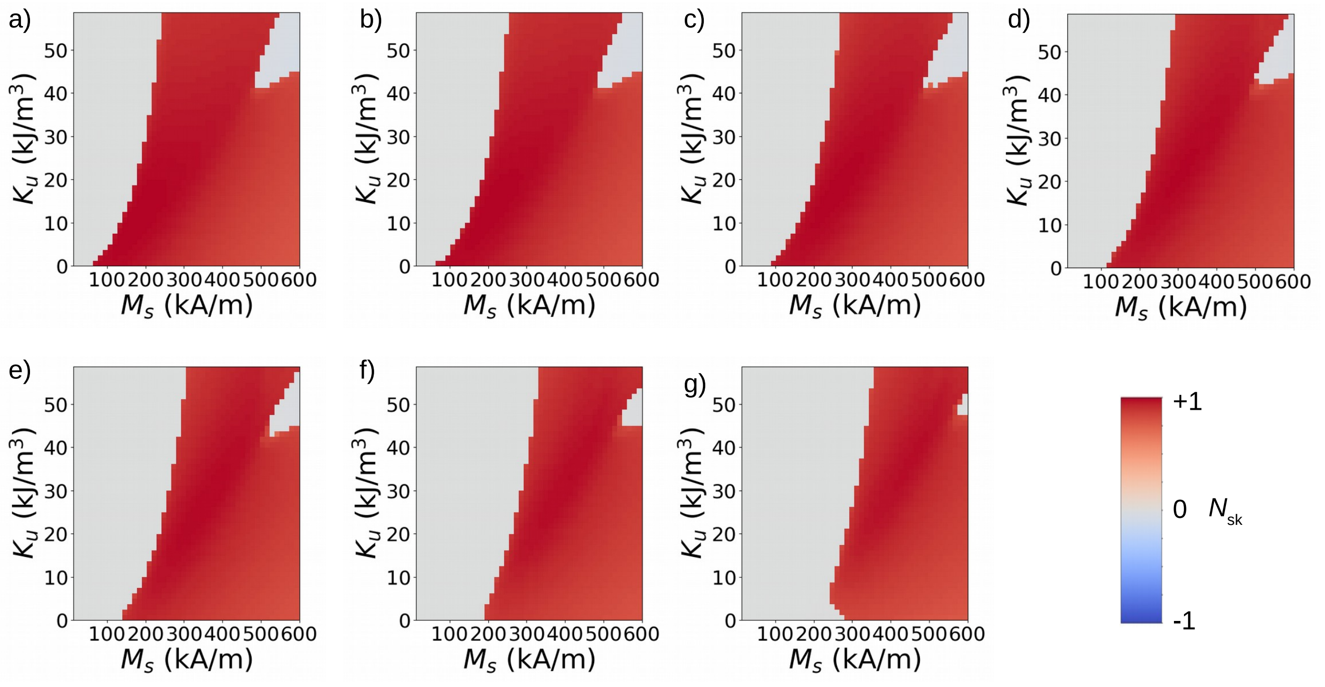

Fig. 17. **Spin object phase diagrams with an initial skyrmion.** Same as Fig. 16, except, a skyrmion is parametrized as the initial magnetization state and relaxed for 10 ns at high damping $\alpha = 1$. The integer topological charge N_{sk} is 1 for a skyrmion, and 0 for a magnetization state with no topologically protected spin object.

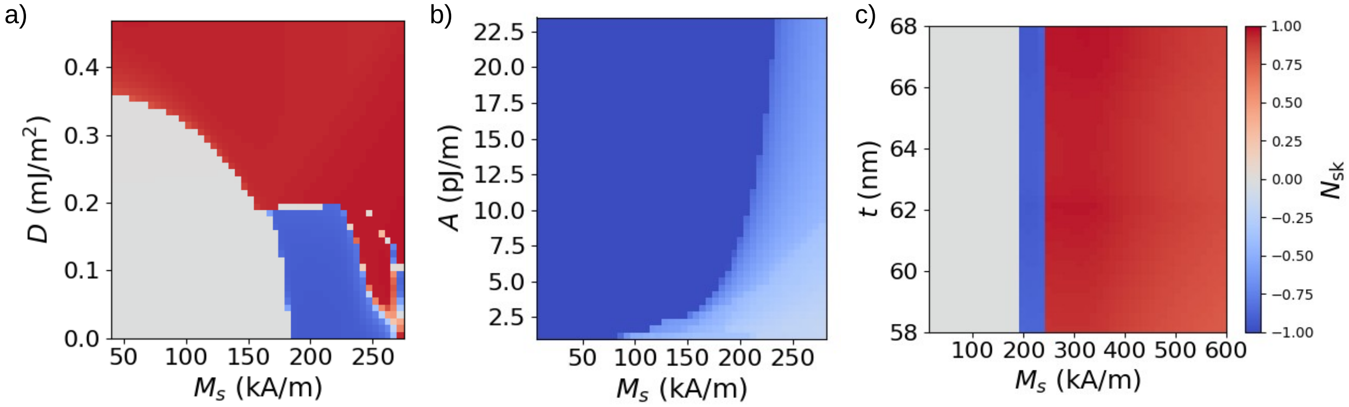

Fig. 18. **Spin object phase diagrams for different material parameters.** Role of material parameters in the stabilization process of an isolated antiskyrmion, as obtained by micromagnetic simulations performed at vanishing fields, where a) shows the influence of the DMI constant D ($1\,\mathrm{mJ/m^2} \sim 340\,\mu\mathrm{eV/atom}$ [5]), b) the exchange stiffness constant A, and c) the thickness t of the film. An antiskyrmion was initially parametrized in the system and then relaxed for 10 ns at high damping $\alpha = 1$. The integer topological charge N_{sk} is -1 for an antiskyrmion, 1 for a skyrmion, and 0 for a magnetization state with no topologically protected spin object.

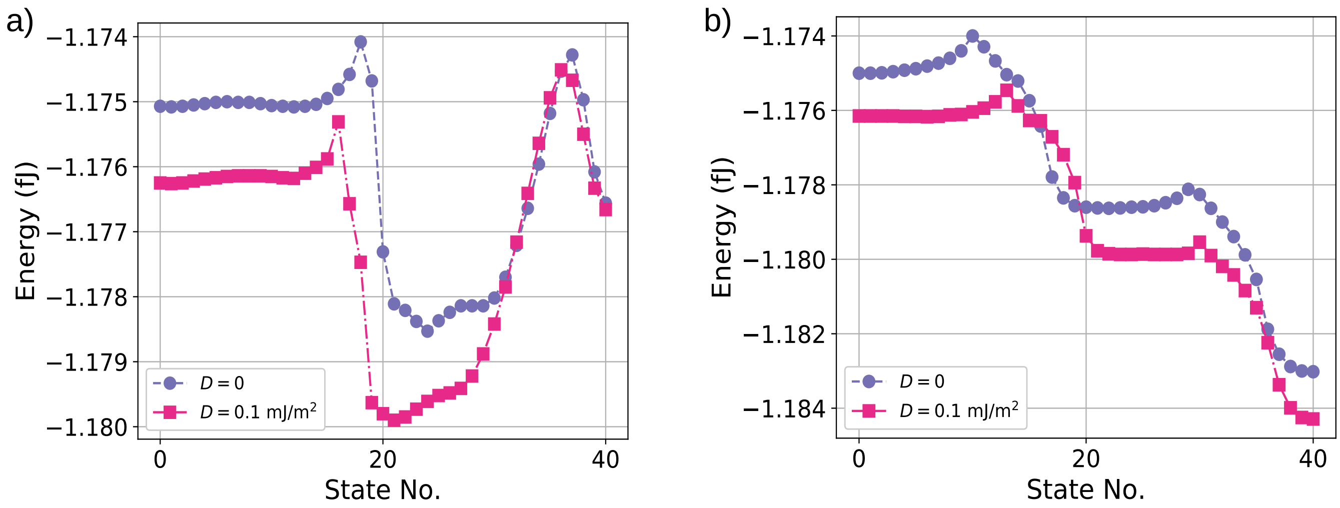

Fig. 19. **Energy paths.** Minimum energy paths for a) the annihilation of an antiskyrmion and its transition to a saturated state along $e_z = (0,\ 0,\ 1)$, and b) for the transition of an antiskyrmion to a Bloch skyrmion.
